# *Minds That Matter:* 2007 Gairdner International Awards Lectures

**Published:** 2007-12

**Authors:** F. Krasnoshtein, N. Nikolov

**Keywords:** Gairdner symposium, antibiotic resistance, cohesin, breast cancer, metastatic, targeted therapy, her2 receptor, vegf, angiogenesis, acute myeloid leukemia, chromosome, translocation, hpv vaccine, apoptosis, cancer stem cells, angiogenic factors, genome search, genome projects, genomic profile, tumour phenotype, programmed cell death

## Abstract

On October 25 and 26, 2007, at the University of Toronto, the Gairdner Foundation in partnership with Canadian Institutes of Health Research presented a two-day international symposium titled *Minds That Matter.* The symposium featured academic lectures by Gairdner Award winners past and present and by other leading biomedical scientists. These distinguished researchers share many characteristics in common: creativity, vision, tenacity, and driving curiosity to illuminate discovery with high degree of relevance. The present article summarizes the 2007 Gairdner Award lectures.

## 1. INTRODUCTION

The year 2007 marked the 48th presentation of the prestigious Gairdner Awards. Among the 288 past recipients of these awards, 70 individuals went on to win a Nobel Prize. Two of those Nobel Laureates presented at the 2007 symposium, *Minds That Matter.* All of the 2007 Gairdner Award lectures were given at the MacLeod Auditorium, Medical Sciences Building, on the University of Toronto campus in downtown Toronto. In the true spirit of the academic community, attendance was open to anyone and was free of charge.

## 2. THE LECTURES

Welcoming remarks by Dr. John Dirks, President of the Gairdner Foundation and chair of the morning session, and by Dr. Catharine Whiteside, Dean, Faculty of Medicine, University of Toronto, launched Day 1 of the symposium.

### 2.1 Day 1, Morning

***Dr. C. David Allis,*** Gairdner Laureate 2007 and Joy & Jack Fishman Professor, The Rockefeller University, New York, New York, kicked off the scientific lectures with “Beyond the double helix: reading and writing the ‘histone code’.” Allis proposed the “histone code hypothesis,” a universal mechanism for modifications in histone proteins that affect the stability of the genome and gene transcription. Moving beyond the major assumption in biomedicine that genes determine disease, Allis turned to epigenetics, the inheritance of phenotype differences not based on changes in dna sequence, by asking if more could be at play than just a genetic blueprint. The answers came from his work on posttranslational modifications of histones, linking histones to cancer by demonstrating that “cancer-related proteins ‘write and read’ histone methyl marks,” thus balancing gene expression. This knowledge changes the scientific understanding of the Watson–Crick double helix by demonstrating that the posttranslational modification of histones has cancer implications—a mechanism that points beyond humanity’s genetic blueprint. To date, several “drug-able” targets aiming at the epigenome have been identified. Applying knowledge of cancer epigenetics, researchers are designing new targeted therapies that halt the gene regulation by posttranslationally modified histones that can ultimately result in aberrant cell growth and differentiation leading to cancer.

Continuing with the genome theme, ***Dr. Kim Nasmyth,*** Gairdner Laureate 2007 and Whitley Professor of Biochemistry, University of Oxford, Oxford, U.K., presented a series of his discoveries relating to mechanisms in cell division in “Protein rings that bind dnas together—a new principle of chromosome organization essential for life?” Sister chromatid cohesion is essential for mitosis, resulting in a “tug of war,” because the chromatids do not easily separate. Nasmyth examined the mechanism by which a cell ensures that sister chromatids move to opposite sides of the cell by asking what holds the chromatids together and what triggers the destruction of that bond when they are ready to separate. He demonstrated that the bond is created by a multi-subunit protein complex called cohesin, a “molecular glue” that holds chromosomes together. Cohesin, a heterodimer, circularizes to form a gigantic ring structure that may hold sister chromatids together by embracing them topographically. As the cohesin dimerization domain twists open, it acts as a receptor to bind dna, and as the dna enters via the cohesin hinge, the cohesin ring shuts, trapping sister chromatids in its midst. Conversely, the carefully regulated protein separase destroys sister chromatid cohesion by cleaving one of the cohesin subunits. This knowledge has therapeutic implications in cancer treatment, because drug targets can now be designed to prevent opening of the cohesin ring, thus inhibiting aberrant cell division and proliferation. Similar applications are possible for other diseases, such as a severe form of the developmental defect Cornelia de Lange syndrome, caused by a mutation in the cohesin protein (Smc), demonstrating that cohesin’s role goes beyond mitotic function.

Shifting focus from the genome, ***Dr. Harry Noller,*** Gairdner Laureate 2007 and Professor of Molecular Biology, University of California, Santa Cruz, California, offered a glimpse of ribosomes in action using biophysical methods in “Ribosome structure and dynamics: caught in the act.” Referring to the ribosome as a “molecular machine,” Noller described structural dynamics of the ribosome during transcriptional movement (translocation) of trna through the ribosome. Using fluorescence resonance energy transfer analysis, he directly observed the intersubunit movement inside a single ribosome by detecting changes in fluorescence. This mechanical “ratcheting” (rotating) movement between two ribosomal subunits facilitated the movement of trna along the ribosome during protein synthesis. Ribosomes fluctuate spontaneously between the classical and hybrid states in the absence of elongation factor (which promotes the activity of guanosine triphosphate), and thermal energy is sufficient to account for the intersubunit rotation underlying the mechanism of translocation. Translation occurs as a series of translation-and-pause events, dwell times fall into the range 0.5–5 s, and translocation events measure three bases and take place in less than 0.1 s. Better understanding of the translocation of trna through the ribosome can lead to strategies for the design of novel antibiotics against pathogenic bacteria that have evolved a variety of mechanisms of resistance to almost all commonly used antibiotics, leading to a worldwide resurgence in serious illness caused by bacterial infections.

Dr. Noller’s lecture on the structure of ribosome set the stage for “From the structure of the ribosome to the design of drugs” by ***Dr. Thomas A. Steitz,*** Gairdner Laureate 2007 and Sterling Professor of Chemistry, Yale University, New Haven, Connecticut. Knowledge of the structure of the ribosome can be used to design drugs against antibiotic-resistant bacteria (whose “evolution trumps intelligent design,” added Steitz). In his studies, Steitz examined binding of antibiotics to the 50S ribosomal subunit and the mechanisms of resistance. His pioneering work on the structure and function of the large ribosomal subunit demonstrated that bacterial protein synthesis involves an rna-catalyzed reaction step, and he identified a structural basis for targeting the ribosome, laying the foundation for the development of novel antibiotics effective against resistant bacteria. Ribosomes contain many closely spaced sites to which antibiotics can bind, and a “combo-antibiotic” that can bind to various ribosomal sites can potentially be designed, becoming a force in overcoming antibiotic resistance.

### 2.2 Day 1, Afternoon

The focus of the afternoon session, chaired by Dr. Jim Woodgett, Senior Investigator, Director of Research, Samuel Lunenfeld Research Institute, Toronto, Ontario, changed from progress in molecular and cellular biology to advances in the treatment of cancer.

***Dr. Dennis Slamon,*** Gairdner Laureate 2007 and Chief, Division of Hematology/Oncology, David Geffen School of Medicine at the University of California–Los Angeles, Los Angeles, California, opened the session with “Molecular diversity of human breast cancer: biologic and therapeutic implications.” Recognizing that breast cancer is highly heterogeneous, Slamon’s groundbreaking work led to the design of trastuzumab (Herceptin: Genentech, San Francisco, CA, U.S.A.) to treat breast cancer in patients over-expressing her2 receptor and revolutionizing how breast cancer is characterized and treated. As a result, clinicians can pre-select patients with this alteration and treat them with targeted therapy, producing remarkable results. In the anthracycline-based adjuvant setting, treating patients who have her2-positive tumours with trastuzumab improved disease-free and overall survival, but increased cardiotoxicity. In an attempt to eliminate anthracyclines from treatment regimens, new studies focusing on targeted biologic therapies are underway.

Based on the hypothesis that upregulation of vascular endothelial growth factor (vegf) in her2-positive metastatic breast cancer contributes to the aggressive phenotype of this disease subgroup, the “angiogenic switch” modulated by trastuzumab can be exploited in the clinic by combined blockade of these two “linked” pathways. Based on the knowledge that neoangiogenesis is increased in her2-positive tumours, a new targeted therapy that combines two biologics, trastuzumab and the anti-vegf antibody bevacizumab (Avastin: Genentech), was tested in the absence of anthracyclines in first-line metastatic breast cancer. In phase i and ii clinical trials, this drug combination demonstrated no untoward toxicity and resulted in a 53% overall response rate and an 86% clinical benefit, setting the stage for phase iii trial. Using specificity of this kind, other cancers could possibly be identified and treated in similar ways. As the genetic information that drives tumours is identified, better outcomes are predicted with less cytotoxic therapies.

A pioneer in work on chromosome translocation, ***Dr. Janet Rowley,*** Gairdner Laureate 1996 and Blum–Riese Distinguished Service Professor of Medicine, Molecular Genetics and Cell Biology and Human Genetics, University of Chicago, Chicago, Illinois, presented “Gene expression in acute myeloid leukemia.” Focusing on chromosome abnormalities in cancer, Rowley discussed how aberrant karyotypes lead to malignancies and how understanding the molecular changes in tumours can lead to more success in treating them. Rowley discovered the Philadelphia chromosome, the first recognized chromosomal abnormality caused by a translocation between the long arms of chromosomes 9 and 22, t(9;22), and resulting in chronic myeloid leukemia (cml). Today, several chromosome translocations have been identified in acute myeloid leukemia (aml), and breakpoints have been cloned. Rowley described the current model of the microrna (mir) mechanism, and how mir analysis can be used as a diagnostic tool to track translocations in aml samples. Differential expression of four mirs in aml as compared with acute lymphoblastic leukemia allows for those two leukemias to be distinguished, diagnosed, and appropriately treated. Future challenges involve identifying additional genetic changes that collaborate with each translocation and developing a more effective therapy targeted to the genes involved in each translocation and to the collaborators. Instead of studying one gene or pathway at a time, which may give a distorted and incomplete view of the cell, a more comprehensive approach that simultaneously analyzes multiple genes and pathways is required.

Continuing with the theme of leukemias started by Rowley, ***Dr. Brian Druker,*** Howard Hughes Medical Institute, Professor of Medicine, Oregon Health and Science University, Portland, Oregon, focused on “Imatinib (Gleevec) as a paradigm of targeted cancer therapies”—an example of how structural analysis of protein can guide drug research. In a Philadelphia chromosome, the t(9;22) translocation brings together two genes: the *BCR* (breakpoint cluster region) gene on chromosome 22 and the proto-oncogene *ABL* (Ableson leukemia virus) on chromosome 9, resulting in a hybrid gene *BCR-ABL* that codes for a fusion protein with tyrosine kinase activity, which activates signal transduction pathways, leading to uncontrolled cell proliferation. In cml, *BCR-ABL* was used as a therapeutic target, resulting in development of imatinib mesylate (Gleevec: Novartis Pharmaceuticals, St. Louis, MO, U.S.A.). Imatinib is currently standard therapy in cml, but despite its success, some patients relapse because of mutations in *ABL,* a circumstance that has led to the development of the novel *ABL* inhibitors nilotinib (AMN107) and dasatinib (Sprycel: Bristol–Myers Squibb, Princeton, NJ, U.S.A.), which are more potent than imatinib. Druker noted that one of the most important lessons learned from his research is that “it’s all about the target!” Early treatment of disease by identifying the right target and matching the right patient to the right drug can have a positive effect on the patient’s disease-free survival and overall survival after a diagnosis of cancer.

Continuing with targeted therapy, ***Dr. Napoleone Ferrara,*** Genetech Fellow, Staff Scientist, San Francisco, California, presented “Anti-angiogenic therapy for cancer and other disorders.” Ferrara described a series of experiments in which inhibition of vegf-mediated angiogenesis resulted in suppression of tumour growth *in vivo,* which led to the development of the recombinant humanized monoclonal anti-vegf antibody bevacizumab. Bevacizumab was the first biologic that demonstrated survival advantage in patients with various tumours that did not respond to standard therapy. In the brite (Bevacizumab Regimen—Investigation of Treatment Effect) trial, the addition of bevacizumab to a variety of chemotherapy regimens improved progression-free survival in metastatic colorectal cancer with overall survival of 91% at 6 months and 77% at 12 months. Angiogenesis also contributes to the pathologic state in age-related macular degeneration, in which blood vessel growth leads to development of lesions. Ranibizumab injection (Lucentis: Genentech) was developed for the treatment of neovascular age-related macular degeneration and was approved by the U.S. Food and Drug Administration (fda) in 2006 based on data from two large, pivotal phase iii clinical trials in which patients demonstrated improvement in visual acuity and remained stable after two years of treatment.

Targeting vegf appears to result in clinical benefit beyond cancer. Other studies have demonstrated that bone marrow cells participate in the regulation of tumour angiogenesis, and tumours less responsive to anti-vegf-a monotherapy recruit more bone marrow cells, suggesting that the tumour primes the bone marrow and instructs it to become angiogenic. Elucidating alternative angiogenic mechanisms may therefore broaden the therapeutic targets for treatment of cancers and other diseases. In physiologic angiogenesis, vegf is a key regulator, and targeting vegf is now a validated strategy for the treatment of several malignancies. Optimizing duration of treatment and combinations with other inhibitors of angiogenesis may enhance the clinical effectiveness of vegf inhibitors.

In “HPV vaccines and the prevention of genital cancers,” ***Dr. Douglas R. Lowy,*** Laboratory of Cellular Oncology, Division of Basic Sciences, National Cancer Institute, National Institutes of Health, Bethesda, Maryland, presented an overview of human papillomavirus (hpv)–induced disease, with cervical cancer being second to breast cancer as the most common cancer in women worldwide. Nearly all cases of cervical cancer (20% in women worldwide, and 80% in the developing world) and other mucosal, cutaneous, and oral cancers stem from hpv infection. Designed by choosing an appropriate molecular target (L1 capsid protein), two distinct HPVL1 vaccines are now being marketed:

- Gardasil (Merck and Co., Whitehouse Station, NJ, U.S.A.), approved in 2006 in Canada and the United States (women aged 9–26 years) and in the European Union (women aged 9–26 years and boys aged 9–15 years); and
- Cervarix (GlaxoSmithKline, Philadelphia, PA, U.S.A.), approved in 2007 in the European Union (women aged 10–25 years) and filed with U.S. fda in March 2007.

Although the vaccine is not a treatment or cure for cervical cancer, it has demonstrated efficacy in preventing infection by specific types of hpv if administered to girls before the start of sexual activity. Outstanding medical issues remain: Will the vaccine continue to have an excellent safety and efficacy profile after it has been given to thousands of people? How long will the vaccine remain protective? Will booster vaccinations be needed? Will the vaccine be effective in boys and men? And might the eradicated hpv types be replaced by other hpv types?

The current HPVL1 vaccines can reduce the incidence of benign and malignant genital hpv infections, but their type-restricted protection means that some serious infections will still occur in vaccinated women. And it is unclear when the hpv vaccine will be widely implemented in the developing world, where most cases of cervical cancer occur. The need for second-generation vaccines is widely recognized.

### 2.3 Day 2, Morning

The morning session of Day 2 of the symposium drew a capacity crowd, welcomed by Dr. John Dirks, President, The Gairdner Foundation, and Dr. Phil Branton, Director, Cancer Institute, Canadian Institutes of Health Research, Ottawa, Ontario. “Advances in our understanding of cancer” promised to be the underlying theme of the day.

From his work on a small nematode, ***Dr. Robert Horvitz,*** Gairdner Laureate 1999, Nobel Laureate 2002, and Professor of Biology and Investigator, Howard Hughes Medical Institute, Cambridge, Massachusetts, showed how misregulation of programmed cell death (apoptosis), a process that occurs during normal development, can contribute to human disease, particularly cancer. Too much cell death can lead to neurodegenerative disease, cerebral stroke, traumatic brain injury, aids, myocardial infarction, congestive heart failure, acute liver injury, aplastic anemia, and sepsis. However, too little cell death can lead to cancer, autoimmune disease, and viral infections. In “Genetic control of programmed cell death in *[Caenorhabditis] elegans*”, Horvitz provided an overview of the core molecular genetic pathway of apoptosis. According to Horvitz, programmed cell death involves four major steps:

1. Identify the victim.
2. Kill.
3. Get rid of the corpse.
4. Destroy the evidence.

Because programmed cell death requires the function of specific genes, it is an active, biologic process; and because Ced3 and Ced4 act within cells that are going to die, programmed cell death is to at least some extent a process of cellular suicide. The *C. elegans* Ced3 looks like interleukin-1β–converting enzyme, a protease implicated in human inflammatory disease, and it can cause mammalian cells to undergo apoptosis. Human Apaf1, which promotes apoptosis in a cell-free system, is similar to Ced4. Although *CED3* (whose protein product acts like a caspase) and *CED4* are “killer” genes, *CED9* is an anti–cell death gene that looks like *BCL2,* a human cancer gene that causes follicular lymphoma. And because human Bcl2 can substitute for nematode Ced9 in *C. elegans,* Ced9 and Bcl2 must act in molecular genetic pathways that are the same or similar. Because caspases kill, caspase inhibition could protect against apoptosis in the treatment of disease with too much cell death. Conversely, because the Ced9 and Bcl2 proteins are protective, their inhibition could promote apoptosis in the treatment of diseases with too little cell death. Knowing the genetic pathways and the genes and proteins involved in apoptosis can lead to the identification of therapeutic targets for specific new drugs for cancer and other diseases.

Examining the “Mechanisms of metastatic spread,” ***Dr. Robert Weinberg,*** Gairdner Laureate 1992 and Professor of Biology and Member, White-head Institute, MIT, Cambridge, Massachusetts, demonstrated that, during the normal differentiation process, cell origin determines metastatic potential. Whether a tumour arises and metastasizes is determined by a subset of cells called “tumour-initiating cells” or “cancer stem cells.” A self-renewing stem cell has the capability to seed a new tumour, but when it enters a differentiation pathway, becoming a “transit amplifying” cell, it loses its tumour-inducing capability. Studies have demonstrated that successful colonization is likely to depend on the self-renewal capacity of disseminated micrometastatic cells and their adaptation to novel microenvironments. To show how cancer cells acquire those capabilities, Weinberg described a series of experiments that demonstrated the “metastatic education” of breast cancer cells (bccs) by signals released from mesenchymal stem cells recruited to tumour-associated stroma. The bccs used an unknown signal to stimulate the recruited mesenchymal stem cells, which responded by releasing lymphoid tissue chemokine, CCL5, to which the bccs responded with increased motility and invasiveness. Said Weinberg, “The nature of the cell-of-origin is a strong determinant of the phenotype of the tumorigenic cell, including its eventual tendency to metastasize,” emphasizing that cell origin is important, and a pre-existing normal differentiation of cell origin determines metastatic phenotype.

In “Cancer stem cells,” ***Dr. John Dick,*** Professor, Medical Genetics and Microbiology, and Senior Scientist, Division of Cellular and Molecular Biology, Toronto General Research Institute, Toronto, Ontario, discussed how an understanding of stem cells in the leukemic process can be harnessed for therapeutic purposes. By asking which cell in cancer has the capability to allow cancer to continue to proliferate, and using *in vivo* repopulation assay in nonobese diabetic/severe combined immunodeficient mice, Dick identified a small subset of stem cells that can initiate leukemia. In aml, only leukemic stem cells can initiate the disease, and little understanding has been attained regarding how normal cells become transformed in the initiation of leukemia. Using the severe combined immunodeficient mouse model to study functional heterogeneity in the hematopoietic system, to characterize the developmental pathways of both cell types, and to understand how the types differ can lead to the elucidation of the mechanism by which the leukemic process alters the development of the normal blood system. A cancer stem cell hierarchy model demonstrated functional heterogeneity within tumours, identifying a subset of self-renewing cells from bulk cancer that are responsible for initiating and maintaining the disease clone. This model has been demonstrated in a variety of cancers, including breast (in 2003), brain (2004), colon (2006), and aml (1994). These findings have therapeutic implications: the elimination of the bulk tumour population may not eradicate cancer stem cells, because those cells may be dormant and drug-resistant, may use cancer-specific pathways differently, or may have altered niche requirements and altered migratory properties. Insights into how the normal and leukemic blood systems differ in their molecular pathways will permit development of effective anti-leukemia therapies that target leukemic stem cells and disrupt the molecular process that leads to leukemia, and of ways to prevent the disease from arising.

***Dr. Tak Mak,*** Gairdner Laureate 1989, Professor and Director, The Campbell Family Institute for Breast Cancer, and Senior Scientist, Division of Stem Cell and Developmental Biology, Advanced Medical Discovery Institute/Ontario Cancer Institute, Toronto, Ontario, opened his philosophical talk “Did the oncogene revolution set back clinical oncology?” by saying that, “in 1976, the oncogene revolution started and it is still going on.” Oncogene research was stalled for a time, and—given popular belief that “what is not too obvious must not be good”—oncogenes were forgotten. Yet, oncogenes are important players in cancer because of their induction of cell proliferation and apoptosis. Survival signals (phosphoinositide-3–kinase, Bcl2, nuclear factor κB) block oncogene-induced cell death, which makes survival and death pathways ideal targets for therapeutic interventions. Imatinib mesylate, an inhibitor of cell survival, is a good example. Drugs that target the survival signals that block oncogene-induced apoptosis can help in the treatment of some cancers. Members of the Rho family are important in metastasis, because they are involved in motility and invasiveness. In certain cells, RhoA transforms and enhances invasiveness, and overexpression of RhoC increases angiogenic factors in breast cells *in vitro* and stimulates melanoma cells to exit the blood and colonize lungs. Furthermore, oncogenes affect metabolism in a profound way by allowing some cancers to proliferate in low glucose conditions by deriving energy from fatty acids. Targeting cell metabolism to starve cancer by aiming at the genes involved in alternative energy sources may have therapeutic implications as an anticancer strategy and could be the dawn of a new era in cancer treatment.

### 2.4 Day 2, Afternoon

The afternoon session of Day 2, chaired by Dr. Ben Neel, Director, Developmental Biology, Ontario Cancer Institute, Toronto, Ontario, continued with the established theme: advances in the understanding of cancer.

Approaching cancer as a disease of the genome, ***Dr. Tom Hudson,*** President and Scientific Director, Ontario Institute of Cancer Research, Toronto, Ontario, spoke on “Genome variation and cancer.” The landscape of human genome variation suggests that, although most genetic polymorphisms are neutral, some affect phenotype and therefore development of disease. Most of the common genetic variations are shared across populations, and the common variants of interest to geneticists are those that give rise to human disease—that is, common variants equal common disease. However, the universe of common variation in the human genome is small, amounting to 15 million polymorphisms. Hudson described several genome projects in which genome-wide searches are used to map polymorphisms and to look for predictors of disease. The goal of the International HapMap Project is to describe common patterns of sequence variation (haplotypes) in the human genome. Genome-wide searches lead to the identification of new disease targets, which in turn enable the design of screening tools for the early identification of risk factors for diseases (including cancers) and guide the development of appropriate treatments. Genome projects such as the Human Genome, the HapMap, and the Cancer Genome (including Assessment of Risk of Colorectal Tumours in Canada) enable research into the complex nature of disease. The most important contribution of these large-scale projects to science is the generation and transfer of resources, databases, and technologies to the scientific community.

The need for predictors of cancer recurrence led ***Dr. Todd Golub,*** Director, Broad Cancer Program, The Broad Institute, Cambridge, Massachusetts, to develop a method of genomic profiling that permits the genetic signature or genetic profile of a tumour to be studied in formalin-fixed tissue. In “Gene expression in cancer,” Golub described three genomic profiles:

- Expression profiles of hepatocellular carcinoma
- Functional genomic profiles of myelodysplasia syndrome
- Drug profiles (“signature-based screening”), and how the use of these profiles in diseases including cancer might help in devising ways to identify compounds of clinical relevance

Examining patterns of recurrence in hepatocellular carcinoma and identifying marker genes led to mapping of a “survival signature” in the liver. This signature revealed some marker genes [for example, antiviral and inflammation-related genes (*MX1, MX2, IRF*s, *IFI*s) and oxidative stress response genes] and overexpressed gene sets (interferon targets, interleukin-6 targets, nuclear factor κB targets) that contribute to a poor prognosis signature, and other marker genes (for example, *CYP*s, *AKR*s, serum proteins) and overexpressed gene sets (for example, liver metabolism) that confer a good prognosis signature. Golub indicated that the signature database provides a connectivity map between the physiologic language of disease, the genetic language of genes, and the organic chemistry language of drugs that can lead to an identification of small molecules of interest with therapeutic potential.

In “Breast cancer genetics and individual risk,” ***Dr. Bruce Ponder,*** Professor of Oncology and Director of Cancer Research, U.K. Cambridge Research Institute, Li Ka Shing Centre, Cambridge, U.K., discussed inherited predisposition in breast cancer and the importance of finding genes that confer susceptibility to cancer. Ponder said that looking at the distribution of risk within a population and studying the genes that accompany that distribution can yield insights into the “genetic architecture” of common cancers, assisting with the design of interventions and screening. Ponder described the use of small nucleotide polymorphisms (snps) as arbitrary markers in genome scans to search for common genetic variants, leading to a correlation of snps with tumour phenotype. Using snps closely linked with tumour phenotypes and genome-wide scanning, candidate gene results from the Breast Cancer Association Consortium (comprising 21 groups worldwide) identified five top gene loci, including *FGFR2* and *MAP3K1.* Based on loci from candidate studies, approximately 5% of genetic variance could be explained, and the identified genes were mostly novel for cancer susceptibility. Ponder concluded that whole-genome association is successful and reproducible in finding common predisposing genes and can be used as a screening tool to uncover individuals at high risk for developing cancer.

Moving from small molecules to worldwide epidemiologic studies, the final lecture of the symposium, “Changing cancer mortality” by ***Dr. Richard Peto,*** Gairdner Laureate 1992 and Professor of Medical Statistics and Epidemiology, University of Oxford, Oxford, U.K., focused on cancer mortality trends. By examining cancer mortality trends attributed and not attributed to tobacco, Peto concluded that smoking matters more than screening, prevention, and treatment in cancer control, although the latter three factors are important in cancer mortality. Among all risk factors, tobacco is the biggest—bigger than chronic infection and occupational and hormonal factors. Worldwide, cancer mortality trends decline when smoking cessation is introduced. From a 50-year prospective U.K. study examining tobacco hazards and mortality trends attributable and not attributable to tobacco, three main messages emerged for the individual smoker:

- The risk is *big*: half of all smokers are killed by their habit.
- A quarter are killed in *middle age* (35–69 years old), losing many life-years.
- *Stopping smoking* works.

If current smoking patterns continue, world tobacco deaths are estimated to reach 1 billion at the end of the 21st century as compared with 0.1 billion for the 20th century. Prevention of a substantial proportion of the 450 million tobacco deaths expected before the year 2050 requires adult smoking cessation.

## 3. CONCLUSION

The two-day *Minds That Matter* academic symposium ended on a high note with closing remarks from Dr. John Dirks, who noted the completion of another successful Gairdner International Awards lecture series by academic leaders in biomedical research whose lifetime of dedication, curiosity, and drive have culminated in unprecedented advances in the understanding and treatment of cancer.

